# Neoadjuvant Chemoradiation in Squamous Cell Carcinoma of the Maxillary Sinus: A 26-Year Experience

**DOI:** 10.1155/2012/413589

**Published:** 2012-09-29

**Authors:** Matthias Kreppel, Sarah Danscheid, Martin Scheer, Jan Christoffer Lüers, Hans Theodor Eich, Joachim E. Zöller, Orlando Guntinas-Lichius, Dirk Beutner

**Affiliations:** ^1^Department of Oral and Cranio-Maxillo and Facial Plastic Surgery, University of Cologne, Kerpener Straße 62, 50931 Cologne, Germany; ^2^Center of Integrated Oncology (CIO) Cologne-Bonn, Kerpener Straße 62, 50931 Cologne, Germany; ^3^Department of Otorhinolaryngology, Head and Neck Surgery, University of Cologne, Kerpener Straße 62, 50931 Cologne, Germany; ^4^Department of Radiotherapy and Radiation Oncology, University of Münster, Albert-Schweitzer-Campus 1, 48149 Münster, Germany; ^5^Department of Otorhinolaryngology, Head and Neck Surgery, Jena University Hospital, Kerpener Straße 62, 50931 Cologne, Germany

## Abstract

*Background*. The aim of our study was to evaluate the effects of neoadjuvant platinum-based radiochemotherapy (RCT) in patients with maxillary sinus squamous cell carcinoma and to compare the results with other multimodality treatment concepts for advanced-stage maxillary sinus carcinoma in the literature. *Methods*. In total, 53 patients with squamous cell carcinoma of the maxillary sinus were reviewed retrospectively. All patients received a neoadjuvant RCT containing either cisplatin or carboplatin followed by radical surgery. Overall survival and locoregional control were plotted by Kaplan-Meier analysis. Prognostic factors were identified through univariate and multivariate analysis. *Results*. Five-year overall survival for all patients was 35%. Eleven patients achieved a complete response after radiochemotherapy. The complete response rate was significantly higher for patients treated with cisplatin (*P* = 0.028); however the 5-year overall survival rates did not differ significantly (*P* = 0.673) for patients treated with cisplatin (37%) and carboplatin (32%). Orbital invasion (*P* = 0.005) and complete response to radiochemotherapy (*P* = 0.021) had a significant impact on overall survival in univariate analysis. *Conclusions*. Neoadjuvant radiochemotherapy followed by radical surgery is an effective treatment for patients with advanced maxillary sinus squamous cell carcinoma. In terms of treatment response cisplatin seems to be more effective than carboplatin.

## 1. Introduction

 Carcinomas of the paranasal sinus are rare, representing 0.2–0.8% of all cancer and 3–5% of the malignant tumors in the head and neck region [1]. The annual incidence is 1-2 per 100 000. The maxillary sinus is the most frequent origin of primary paranasal sinus malignancies [2]. Management of patients with paranasal sinus carcinomas remains a great challenge due to several reasons. Most patients present with a locoregionally advanced disease as the tumor remains asymptomatic for a long time and even early symptoms are similar to common nasal complaints [3]. The complexity of the anatomic site and the histologic heterogeneity leads to difficulties in the classification and staging of paranasal sinus carcinoma [4, 5]. Correct staging however is mandatory as otherwise the patients cannot be assigned to the best treatment scheme for their individual situation, and the treatment effects cannot be evaluated properly [6]. This is particularly important for rare tumors such as maxillary sinus carcinomas as no prospective randomized trials have been conducted and no standardized treatment regime has evolved so far [4, 7]. Therefore, the optimal treatment approach for locally advanced paranasal sinus carcinoma remains controversial.

Only few centers have published treatment results of large groups for patients with paranasal sinus carcinoma, all of them carrying the inherent patient selection bias in retrospective studies, leading to a selection of patients with small resectable tumors [8, 9]. Despite improvements in surgery and radiotherapy (RT) during the last years, patients with advanced stage maxillary sinus carcinoma still have a dismal prognosis, yielding 5-year overall survival rates of 35–49% [10, 11]. Study results suggest that surgery should be incorporated into a treatment regime for patients treated with curative intent [12]. A meta-analysis revealed that disease specific survival after five years is significantly higher for patients treated with surgery plus adjuvant radiotherapy (RT) (66%) than for patients treated with RT alone (46%) [4]. Today, a multimodal treatment regime consisting of surgery, RT, and chemotherapy (CT) is generally applied for patients with advanced-stage head and neck cancers [13, 14]. Several studies have demonstrated that addition of platinum-based CT to adjuvant RT may be beneficial for patients with advanced head neck cancer [15, 16]. The rationale to incorporate CT into multimodal treatment schemes is the synergistic effects with RT, which are the increased tumor cell death through inhibition of the DNA repair in tumor cells, the decrease in tumor mass, the subsequent reoxygenation and radioing of hypoxic tumor cells, and the selective toxicity depending on the cell cycle phase and induction of apoptosis [17]. Concomitant radiochemotherapy (RCT) seems to be more effective than sequential strategies resulting in a survival gain of 6.5% at 5 years. Sequential application of RT and CT yielded worse results [18]. However, the incorporation of platinum analogues is associated with higher neurotoxic side effects [19].

The question of when RCT should be administered—preoperatively or postoperatively—remains open. Several studies have proven the effect and security of neoadjuvant RCT followed by radical surgery in oral squamous cell carcinoma [20–24]. We could demonstrate recently that neoadjuvant RCT is superior to primary surgery followed by adjuvant RCT in patients with oral squamous cell carcinoma with cervical lymph node involvement of stage N2 [25]. Preoperative radiochemotherapy reduces the risk of perioperative tumor cell spreading with implanted metastases, facilitates complete resection, and offers the opportunity of tissue preservation and retained functional integrity [26]. The radiotherapeutic effect is improved in comparison to postoperative radiochemotherapy, due to a higher oxygenation of the tumor [17]. However, this treatment approach can be associated with a higher rate of peri- and postoperative complications [17]. Most centers however advocate primary surgery followed by adjuvant RT or RCT [27]. The major advantages of this concept are the opportunity to obtain a histopathological staging (pTNM) and fewer peri- and postoperative complications.

Neoadjuvant treatment of advanced-stage head and neck cancer has a long tradition at the University of Cologne. Knöbber et al. published promising results on preoperative radiotherapy for patients with oral and oropharyngeal cancer treated at our institutions between 1973 and 1984 [28]. In 1994 results for concomitant cisplatin-based neoadjuvant RCT followed by radical surgery for advanced-stage oral squamous cell carcinoma were published [29]. 

The aim of the present study was to evaluate the effects of neoadjuvant platinum-based RCT in patients with maxillary sinus squamous cell carcinomas in two treatment centers and to compare the results with other multimodality treatment concepts for advanced stage maxillary sinus carcinoma in the literature.

## 2. Material and Methods

### 2.1. Patients

The retrospective study included 53 treatment-naive patients with biopsy-proven primary squamous cell carcinomas of the maxillary sinus, who were treated with curative intent at the Department of Oral and Maxillofacial Plastic Surgery, University of Cologne and at the Department of Otorhinolaryngology and Head Neck Surgery, University of Cologne, between 1980 and 2006. 

In total, there were 187 patients with squamous cell carcinoma of the maxillary sinus, who presented between 1980 and 2006. Distribution of stages was as follows: Stage I: 20 patients, stage II: 18 patients, stage III: 23 patients, stage IVa: 99 patients, stage IVb: 29 patients, and stage IVc: 8 patients.

As this is a retrospective study, an interdisciplinary team of surgeons and radiation oncologists determined the indications for concurrent postoperative RCT individually so that there are patients with stage II, who were chosen for a neoadjuvant treatment, whereas other patients with more advanced stage tumors were not given a neoadjuvant treatment. The patients' clinical characteristics are listed in Table 1. For all patients, clinical and pathologic staging was retrospectively updated to the 7th edition of the UICC for carcinomas of the maxillary sinus [30]. Clinical staging was updated from endoscopy of the upper aerodigestive tract and radiological diagnostic procedures such as CT, MRI, conventional tomography, and sonographic and scintigraphic pretreatment reports. The clinical size of the lymph nodes was determined by B-scan sonography. Lymph nodes of a diameter >1.5 cm were considered as positive [25]. Patients with distant metastases were excluded from our study. 

Clinicopathologic parameters were obtained from the medical charts including the histopathologic and surgical reports. Follow-up data were gathered from a combination of chart reviews and the local government office for registration of residents. 

### 2.2. Treatment

All patients received a concomitant neoadjuvant radiochemotherapy followed by radical surgery. Radiotherapy was delivered by 5-6 MV photons delivered by a linac accelerator in daily fractions of 1.8 Gy five times a week, adding up to a total dose of 39.6 Gy or 50.4 Gy to the primary tumor and to the neck lymph node levels I-V by opposing lateral ports. Supraclavicular nodes were treated in an anterior-posterior field. Carboplatin and cisplatin were administered during the first week of radiotherapy for 5 days as a short-term infusion 1 hour before radiation at a dose of 70 mg/m^2^/day for carboplatin and 40 mg/m^2^/day for cisplatin. Clinical lymph node status of the neck was assessed by B-scan sonography, MRI scan, and computer tomography, respectively. 

Three to four weeks after the end of the neoadjuvant RCT, all patients received a radical modified neck dissection and a radical resection of the primary tumor, either via lateral rhinotomy or the midfacial degloving approach. Three patients, who refused surgery and therefore did not complete the treatment regime, were excluded from our study. A complete response (CR) was defined by histopathology if no viable tumor cells were detectable in the primary tumor as well as in the neck dissection specimen.

### 2.3. Statistical Analysis

Structural differences between groups were assessed using the Wilcoxon rank-sum test for continuously distributed variables and the *χ*² test and Fisher's exact test for categorical variables. The Kaplan-Meier survival analysis method was used to estimate the events of interest for overall survival (OS) and locoregional control (LRC). OS was defined as the time interval from beginning of primary therapy until the patient's death. Patients who did not die were censored at their last date of followup. LRC was defined as the time interval from beginning of primary therapy until locoregional relapse. Patients who did not suffer a locoregional relapse were censored at their last date of follow up [31]. The logrank test was used to compare survival times among patients with different characteristics. *P* values of less than 0.05 were considered as significant and printed in bold. A Cox proportional hazard model with forward selection was calculated for multivariate analysis to estimate the impact of prognostic factors in multivariate analysis [32].

## 3. Results

Table 1 shows the patient and tumor characteristics. At the time of analysis 38 patients were deceased (72%) and 15 were alive. The average and median follow-up times for the patients alive were 98 months and 79 months, respectively. No treatment-related deaths or any cases where patients had to stop the treatment due to toxicity were seen. Radiation in combination with carboplatin was very well tolerated in terms of toxicity and side effects by our patients. 18 patients (34%) suffered a relapse during the followup. 

**Figure 1:**
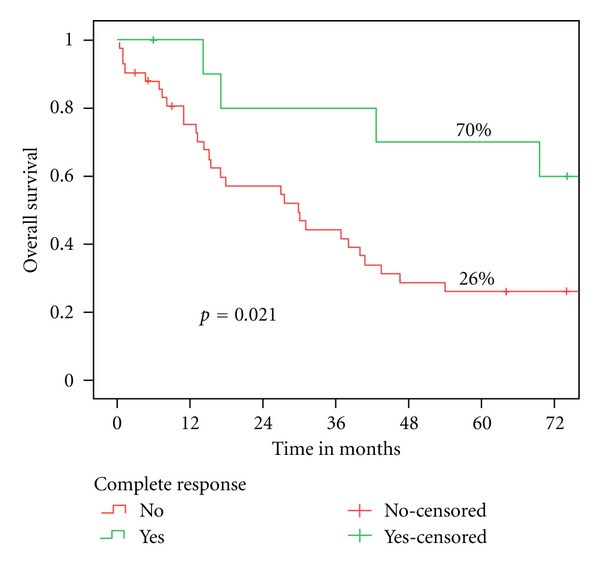
Overall survival according to the response to neoadjuvant RCT.

Table 2 displays the results of the univariate analysis. Orbital invasion of the tumor (*P* = 0.005) had a significant impact on overall survival (*P* = 0.005) and on locoregional control (LRC) (*P* < 0.001). As shown in Figure 1, the patients who achieved a CR had a significantly higher 5-year overall survival rate than patients without CR (70% versus 26%, *P* = 0.021). Patients who received cisplatin had a higher 5-year overall survival rate than patients who were treated with carboplatin (37.2% versus 31.7%); however, the differences observed were not statistically significant (*P* = 0.673). No significant survival differences were observed between the group which received a radiation of 40 Gy and the other group, which received 50 Gy (*P* = 0.501). Clinical staging criteria did not significantly influence overall survival and locoregional control. 

The *χ* ^2^ test revealed that patients who received cisplatin instead of carboplatin had a significantly higher complete response rate after neoadjuvant RCT (*P* = 0.028) (Table 3).

The results of the multivariate analysis are shown in Table 4. Only tumor infiltration of the orbita had a significant impact on overall survival in multivariate analysis (*P* = 0.012). Patients who achieved a CR had a smaller relative risk (RR) of death (0.463) than patients with residual tumor after neoadjuvant RCT; however, the differences were not statistically significant (*P* = 0.157). 

## 4. Discussion

The main goals in treating paranasal sinus cancer are to cure the cancer, preserve or restore the facial contour and function, minimize the sequelae of treatment, and prevent secondary tumors. However, in patients with advanced tumors these targets are rarely achieved. The objective of this study was to investigate the treatment outcome of platinum-based neoadjuvant RCT followed by radical surgery in patients with squamous cell carcinoma of the maxillary sinus and to compare the results with other multimodal treatment regimes published. The 5-year overall survival rate of all patients was 35%. Considering the relative rarity of the disease, the various histological types, and the different possible sites of origin, we managed to gather homogeneous study group of the considerable size of 53 patients. 

Numerous studies have shown that a combined modality treatment is required for tumors beyond stage II [33–37]. Sole surgical treatment of advanced tumors leads to a significantly reduced overall survival and locoregional control whereas definitive RT or RCT rarely results in a complete remission and subsequent cure of the tumor [12, 38]. Apart from that, many patients treated with definitive RT at doses of 65 Gy and more suffer from visual impairment. About 20–30% of the patients develop ipsilateral blindness due to optic neuropathy and 10–20% lose their eyesight bilaterally, which seems to be due chiasm injury [39, 40]. 

In our study, 18 patients received a radiation dose of 40 Gy and 35 patients received 50 Gy. Isobe et al. used a preoperative median radiation dose of 60 Gy ranging from 26–76 Gy in combination with 5-fluorouracil or peplomycin, resulting in a 5-year overall survival rate of 57% [41]. They concluded that the total dose of the radiation and the cumulative dose of the chemotherapy did not have a significant influence on the outcome. Ashraf et al. used a dose of 50 Gy for patients receiving preoperative radiotherapy for cancer of the maxillary sinus [42]. The 5-year survival rate for these patient subgroups was 47%. But it has to be taken into account that most of the patients had T3 tumors and not T4 tumor like in our study sample. At our institution we applied 40–50 Gy in a preoperative setting for head and neck squamous cell carcinoma. This dose results in a mild toxicity [20, 43] and was developed from the experience of previous studies carried out at our department [28, 29]. However, there are no studies that compare the effects of different radiation doses in a neoadjuvant setting. 

However, the “ideal combination” of the different treatment modalities of surgery RT and CT is still under debate. The first study conducted on this matter could not detect any significant differences between preoperative and postoperative irradiation [44]. 

Only one study could detect a significant difference between pre- and postoperative RT. Hu et al. found a significantly higher 5-year overall survival for patients treated with preoperative RT (64%) than for patients treated with postoperative RT (26%) [45]. However, no CT was administered during treatment. A meta-analysis of all kinds of head and neck cancers reported an increase of overall survival of 4.5% at 5 years for all patients who received CT in addition to RT. For patients who were treated in a concomitant setting the survival gain was even higher (6.5%) [46]. In 2002, Nibu et al. published a study on 25 patients with squamous cell carcinoma of the maxillary antrum treated with neoadjuvant RCT consisting of 30–40 Gy and intraarterial infusion of cisplatin in combination with 5-FU. For eight patients with skull base invasion, the CT was administered intravenously. In this study an excellent 5-year overall survival rate of 72% was achieved [36]. Madison et al. used the RADPLAT protocol to treat patients with advanced paranasal sinus carcinoma. Patients received four intraarterial infusions of cisplatin and 50 Gy in a neoadjuvant concomitant setting [47]. Six weeks after the end of the RCT, a radical surgery was performed via a craniofacial approach. The 5-year overall survival rate was 81%. However, there are some limitations to the study as the total number of patients was only 11 with none of the patients having cervical lymph node metastases. The latter is of importance as cervical lymph node metastases are considered to be one of most adverse prognostic factors [48]. The incidence of cervical lymph node metastases is reported to be 10–20% [40, 49]. In our study, 47% of the patients exhibited cervical lymph node metastases at the time of diagnosis. A possible explanation for this relatively high percentage is that the patients at our institutions treated with neoadjuvant RCT and radical surgery were patients considered to be at a high risk level with an unresectable disease. This might explain the lower overall survival rate of 35% at five years in our treatment group. 

Eleven patients (21%) achieved a CR through the neoadjuvant RCT. Some studies that examined neoadjuvant RCT in patients with paranasal sinus carcinoma did not provide any information on the response [36, 45]. Hanna et al. reported a partial reponse rate of 67% after neoadjuvant induction chemotherapy consisting of a platinum derivate and a taxane or a combination with a third agent, such as ifosfamide or 5-fluorouracil. They found that response to the induction chemotherapy but not the subsequent local therapy (surgery, definitive radiotherapy, or radiochemotherapy) was predictive for overall survival of the patients [50]. Patients who had no histopathological sign of a residual vital tumor after neoadjuvant RCT had a 5-year overall survival rate of 70%. Patients with incomplete response or nonresponse had a significantly lower 5-year overall survival rate of 26% (*P* = 0.021). Papadimitrakopoulou et al. reported a complete response rate of 26% after a CT with intraarterial cisplatin application and intravenous application of ifosfamide and paclitaxel [51]. However, the response was assessed radiologically, which does not allow the detection of microscopic residual disease. This concept carried substantial toxic side effects such as cerebrovascular ischemia and cranial neuropathia, which were not observed in our study. Another promising multimodality treatment scheme was published in 1999: 19 Patients with stage III or IV disease were treated with induction chemotherapy followed by surgery and postoperative concomitant radiochemotherapy resulting in a clinical response of 87% and an impressive 5-year overall survival rate of 72.7%. Chemotherapy consisted of three cycles of cisplatinand 5-fluorouracil [52]. Samant et al. treated 19 patients with neoadjuvant radiation therapy with a total dose of 50 Gy in 5 weeks in combination with 3-4 weekly intra-arterial infusions of infusions of cisplatin followed by surgery six weeks after the end of the neoadjuvant treatment. There were no toxic side effects that limited the treatment. They achieved a 5-year overall survival rate of 53% despite the advanced stage of disease (16 patients with T4-disease, 3 patients with T3-disease) [53].

A major problem in analyzing the treatment results for patients with neoadjuvant RCT is that no pTNM is available. As a consequence, meticulous clinical examinations including imaging techniques such as CT, MRI, PET, and endoscopic exploration of the upper aerodigestive tract are required in order to provide detailed and reliable staging information. Although pathological staging is regarded superior to clinical staging in head neck cancer [54], a recent study demonstrated that the prognostic value of clinical staging is equal to pathologic staging [55]. 

In our study, patients treated with cisplatin had a significantly higher complete response rate than patients treated with carboplatin (*P* = 0.028). Out of 11 patients with complete response, 10 patients received cisplatin instead of carboplatin. Cisplatin is generally preferred to carboplatin in most centers and seems to yield higher response rates in various types of squamous cell head and neck cancer [21, 24, 56–58]. Two prospective studies compared the effects of cisplatin and carboplatin. Both studies demonstrated advantages for cisplatin over carboplatin [59, 60]. However, the settings in both studies substantially differed from our study. In the study of the Southwest Oncology Group (SWOG), comprising 277 patients with carcinoma from all different sites of the head and neck, the effects of cisplatin and 5-FU versus carboplatin and 5-FU were compared. Despite a higher response rate in the cisplatin group (32% versus 21%) survival was similar for both groups. However, ototoxicity and renal and hematologic toxic effects were substantially greater in the cisplatin group [60]. The other study found a significantly higher response rate for patients with different head and neck tumors treated with cisplatin in comparison to carboplatin. However, the CT was administered as an induction CT and not concomitant [59]. A prospective randomized trial comparing the effects in a concomitant setting with RT is still missing. 

In summary, our study results indicate that neoadjuvant radiochemotherapy followed by radical surgery is an option for patients with locoregionally advanced maxillary sinus squamous cell carcinoma. Patients who achieve a complete response after neoadjuvant RCT have a very good prognosis despite the advanced tumor stage; however, patients who do not respond still have a dismal prognosis. In terms of treatment response, cisplatin seems to be more effective than carboplatin for these tumors.

## Figures and Tables

**Table 1:** Patient and tumor characteristics.

Patients* n *	53
Age (years)	
Mean ± standard deviation	57.9 ± 11.3
Median	58.0
Minimum/maximum	18/78
Gender *n* (%)	
Male	41 (77.4%)
Female	12 (22.6%)
cT-classification *n* (%)	
T2	3 (5.7%)
T3	11 (20.8%)
T4a	34 (64.1%)
T4b	5 (9.4%)
cN-classification *n* (%)	
N0	28 (52.8%)
N1	6 (11.3%)
N2	19 (35.8%)
UICC stage grouping *n* (%)	
II	2 (3.8%)
III	10 (18.9%)
IVa	36 (67.9%)
IVb	5 (9.4%)
Complete response (CR) *n* (%)	
No	42 (79.2%)
Yes	11 (20.8%)
Orbital infiltration *n* (%)	
No	11 (20.8%)
Yes	42 (79.2%)
Relapse *n *(%)	
No	35 (66.0%)
Yes	18 (34.0%)
Radiotherapy *n *(%)	
40 Gy	18 (34.0%)
50 Gy	35 (66.0%)
Chemotherapy *n *(%)	
Carboplatin	20 (37.7%)
Cisplatin	33 (62.3%)

**Table 2:** Univariate analysis of prognostic factors.

Variable	5-year OS	*P *value	5-year LRC	*P *value
All patients	35.0%		58.6%	
Age		0.089		0.627
≤58 years (lower half of median)	43.9%		63.9%	
>58 years (upper half of median)	26.9%		52.1%	
Gender		0.178		0.951
Male	30.3%		60.1%	
Female	50.0%		56.3%	
cT-classification		0.497		0.135
T2	33.3%		100%	
T3	30.3%		70.0%	
T4a	39.1%		56.1%	
T4b	20.0%		25.0%	
cN-classification		0.834		0.235
N0	37.2%		64.0%	
N1	53.3%		100%	
N2	28.1%		45.3%	
UICC stage grouping		0.228		0.191
II	50.0%		100%	
III	22.5%		64.3%	
IVa	39.7%		59.0%	
IVb	20.0%		25.0%	
Complete response (CR)		**0.021**		0.136
No	26.0%		52.0%	
Yes	70.0%		78.8%	
Orbital infiltration		**0.005**		**<0.001**
No	44.0%		23.3%	
Yes	0%		68.2%	
Radiotherapy		0.501		0.950
40 Gy	41.7%		58.9%	
50 Gy	31.3%		57.8%	
Chemotherapy		0.673		0.409
Carboplatin	31.7%		49.4%	
Cisplatin	37.2%		63.9%	

**Table 3:** Association of chemotherapy and complete response (CR).

	No CR	CR	*P* = 0.028
Carboplatin	19	1	
Cisplatin	23	10	

Total	42	11	

**Table 4:** Multivariate analysis of prognostic factors (RR = relative risk; CI = confidence interval).

Variable	Category	*P *value	RR	95% CI
Age	≤58 years versus >58 years	0.292	0.688	0.32–1.41
UICC stage grouping	II and III versus IVa and IVb	0.110	0.540	0.25–1.15
Complete response	CR versus no CR	0.157	0.463	0.16–1.34
Orbital infiltration	No versus yes	**0.012**	0.348	0.15–0.79
Chemotherapy	Cisplatin versus carboplatin	0.957	0.979	0.46–2.10
